# Morphological and molecular identification of four new resupinate species of *Lyomyces* (Hymenochaetales) from southern China

**DOI:** 10.3897/mycokeys.65.48660

**Published:** 2020-03-26

**Authors:** Jun-Zhu Chen, Chang-Lin Zhao

**Affiliations:** 1 Key Laboratory for Forest Resources Conservation and Utilization in the Southwest Mountains of China, Ministry of Education, Southwest Forestry University, Kunming 650224, China; 2 College of Biodiversity Conservation, Southwest Forestry University, Kunming 650224, China; 3 Key Laboratory of Forest Disaster Warning and Control of Yunnan Province, Southwest Forestry University, Kunming 650224, China

**Keywords:** Phylogeny, Schizoporaceae, taxonomy, wood-inhabiting fungi, Yunnan Province

## Abstract

Four new wood-inhabiting fungal species, *Lyomyces bambusinus*, *L. cremeus*, *L. macrosporus* and *L. wuliangshanensis*, are proposed based on a combination of morphological and molecular evidence. *Lyomyces bambusinus* is characterized by resupinate basidiomata with colliculose to tuberculate hymenial surface and broadly ellipsoid, hyaline, slightly thick-walled, smooth basidiospores. *Lyomyces cremeus* is characterised by resupinate basidiomata with smooth, cream hymenial surface and ellipsoid, hyaline, thin-walled to slightly thick-walled basidiospores. *Lyomyces macrosporus* is characterized by pruinose basidiomata with reticulate hymenial surface, presence of three kinds of cystidia and larger basidiospores (6.7–8.9 × 4.4–5.4 µm). *Lyomyces wuliangshanensis* is characterized by coriaceous basidiomata and ellipsoid, hyaline, slightly thick-walled, smooth basidiospores. The phylogenetic analyses based on molecular data of the internal transcribed spacer (ITS) region sequences revealed that the four new species belonged to *Lyomyces*. *Lyomyces bambusinus* grouped with *L. sambuci*. *Lyomyces cremeus* clade was sister to a clade comprised of *L. microfasciculatus*. *Lyomyces macrosporus* was sister to *L. allantosporus*. *Lyomyces wuliangshanensis* was closely related to *L. mascarensis*.

## Introduction

*Lyomyces* P. Karst. typified by *L. sambuci* (Pers.) P. Karst., is a small corticioid genus characterized by resupinate to effused basidiomata with smooth to granular or odontioid hymenophore, a monomitic hyphal system bearing clamp connections, strongly encrusted generative hyphae, the presence of several types of cystidia; clavate to suburniform basidia, and smooth, thin- to slightly thick-walled, cyanophilous basidiospores (Karsten 1881; Bernicchia and Gorjón 2010). The members of *Lyomyces* grow on dead, still-attached or fallen branches of angiosperms, on dead wooden and herbaceous stems, or occasionally on gymnosperm wood (Yurchenko et al. 2017). Twenty-three species are currently known in *Lyomyces* worldwide (Rabenhorst 1851; Karsten 1881, 1882; Peck 1903; Bourdot and Galzin 1911; Cunningham 1959, 1963; Wu 1990; Hjortstam and Ryvarden 2009; Yurchenko et al. 2013, 2017; Gafforov et al. 2017; Riebesehl and Langer 2017) and five species were recorded in China (Xiong et al. 2009; Gafforov et al. 2017; Riebesehl and Langer 2017).

Molecular studies on *Lyomyces* and related genera have been carried out recently (Riebesehl and Langer 2017; Yurchenko et al. 2017; Viner et al. 2018; Riebesehl et al. 2019). Riebesehl and Langer (2017) indicated that *Hyphodontia* s.l. should be divided into several genera: *Hastodontia* (Parmasto) Hjortstam & Ryvarden, *Hyphodontia* J. Erikss, *Kneiffiella* (Pers.) Gray, *Lagarobasidium* Jülich, *Lyomyces* and *Xylodon* (Pers.) Gray and thus 35 new combinations were proposed, including fourteen *Lyomyces* species. The clarification of *Lyomyces sambuci* complex was conducted based on ITS and 28S sequences analyses and four new species of *Lyomyces* were described (Yurchenko et al. 2017). Viner et al. (2018) studied the taxonomy of *Lagarobasidium* and *Xylodon*, and showed that twelve species clustered into *Lyomyces* clade and then grouped with *Xylodon* clade. Phylogenetic and morphological studies on *Xylodon* showed that *Xylodon* was distinct from *Hastodontia*, *Hyphodontia*, *Kneiffiella* and *Lyomyces* and the *Lyomyces* generic species *L. sambuci* was sister to *L. crustosus* (Pers.) P. Karst. formed a single lineage with a high support (Riebesehl et al. 2019).

During investigations on wood-inhabiting fungi in southern China, four additional taxa were found, which could not be assigned to any described species in *Lyomyces*. In this study, the authors expand samplings from previous studies (Gafforov et al. 2017; Riebesehl and Langer 2017) to examine taxonomy and phylogeny of them within *Lyomyces*, based on the internal transcribed spacer (ITS) regions sequences.

## Materials and methods

### Morphological studies

The specimens studied have been deposited in the herbarium of Southwest Forestry University (SWFC), Kunming, Yunnan Province, P.R. China. Special color terms follow Petersen (1996). Macromorphological descriptions are based on field notes. Micromorphological data were obtained from the dried specimens and observed under a light microscope following Dai (2010) and Cui et al. (2019). The following abbreviations are used: KOH = 5% potassium hydroxide; CB = cotton blue; CB+ = cyanophilous; IKI = Melzer’s reagent; IKI– = non-amyloid and non-dextrinoid; L = mean spore length (arithmetic average of all spores); W = mean spore width (arithmetic average of all spores); Q = L/W ratio; n (a/b) = number of spores (a) measured from given number (b) of specimens.

### DNA extraction and sequencing

CTAB rapid plant genome extraction kit-DN14 (Aidlab Biotechnologies Co., Ltd, Beijing) was used to obtain genomic DNA from dried specimens, according to the manufacturer’s instructions (Han et al. 2016; Song and Cui 2017) . The ITS region was amplified with the primer pair ITS5 and ITS4 (White et al. 1990). The PCR cycling procedure for ITS was as follows: initial denaturation at 95 °C for 3 min, followed by 35 cycles at 94 °C for 40 s, 58 °C for 45 s and 72 °C for 1 min, and a final extension of 72 °C for 10 min followed Shen et al. (2019). The PCR products were purified and directly sequenced at Kunming Tsingke Biological Technology Limited Company, Yunnan Province, P.R.China. All newly generated sequences were deposited in GenBank (Table 1).

**Table 1.** List of species, specimens and GenBank accession numbers of sequences used in this study.

Species name	Sample no.	GenBank accession no.	References
		ITS	
*Lyomyces allantosporus*	KAS-GEL 4933	KY800401	Yurchenko et al. 2017
	FR 0249548	KY800397	Yurchenko et al. 2017
*Lyomyces bambusinus*	CLZhao 3675	MN945969	Present study
	CLZhao 4808	MN945970	Present study
	CLZhao 4831	MN945968	Present study
	CLZhao 4840	MN945971	Present study
*Lyomyces cremeus*	CLZhao 2812	MN945973	Present study
	CLZhao 4138	MN945974	Present study
	CLZhao 8295	MN945972	Present study
*Lyomyces crustosus*	YG-G 39	MF382993	Gafforov et al. 2017
	UC 2022841	KP814310	Rosenthal et al. 2017
*Lyomyces erastii*	MA-Fungi 34336	JX857800	Gafforov et al. 2017
	YG 022	MF382992	Gafforov et al. 2017
*Lyomyces griseliniae*	KHL 12971	DQ873651	Larsson et al. 2006
*Lyomyces juniperi*	KAS-GEL 4940	DQ340316	Yurchenko et al. 2017
	FR 0261086	KY081799	Riebesehl and Langer 2017
*Lyomyces macrosporus*	CLZhao 4516	MN945977	Present study
	CLZhao 4531	MN945978	Present study
	CLZhao 8605	MN945975	Present study
	CLZhao 3951	MN945976	Present study
*Lyomyces mascarensis*	KAS-GEL 4833	KY800399	Yurchenko et al. 2017
	KAS-GEL 4908	KY800400	Yurchenko et al. 2017
*Lyomyces microfasciculatus*	CLZhao 4626	MK343568	Present study
	CLZhao 5109	MN954311	Present study
	TNM F 24757	JN129976	Yurchenko and Wu 2014
*Lyomyces organensis*	MSK 7247	KY800403	Yurchenko et al. 2017
*Lyomyces orientalis*	KAS-GEL 3376	DQ340325	Yurchenko et al. 2017
	KAS-GEL 3400	DQ340326	Yurchenko et al. 2017
*Lyomyces pruni*	Ryberg 021018	DQ873624	Larsson et al. 2006
*Lyomyces sambuci*	80 SAMHYP	JX857721	Yurchenko et al. 2017
	83 SAMHYP	JX857720	Yurchenko et al. 2017
*Lyomyces vietnamensis*	TNM F 9073	JX175044	Yurchenko and Wu 2014
*Lyomyces wuliangshanensis*	CLZhao 4108	MN945980	Present study
	CLZhao 4144	MN945981	Present study
	CLZhao 4167	MN945979	Present study
	CLZhao 4206	MN945982	Present study
	CLZhao 4475	MN945983	Present study
*Palifer verecundus*	KHL 12261	DQ873642	Larsson et al. 2006
*Xylodon asperus*	UC 2023169	KP814365	Yurchenko et al. 2017

### Phylogenetic analyses

**Figure 1.**
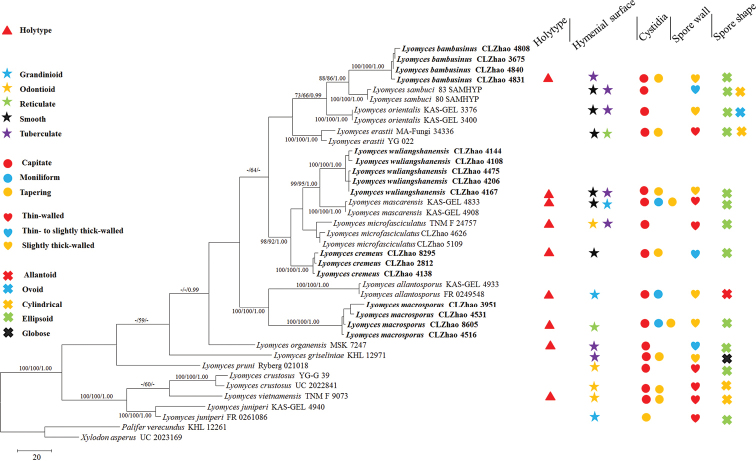
Maximum parsimony strict consensus tree illustrating the phylogeny of four new species and related species in *Lyomyces* based on ITS sequences. Branches are labelled with maximum likelihood bootstrap equal to or higher than 70%, parsimony bootstrap proportions equal to or higher than 50% and Bayesian posterior probabilities equal to or high than 0.95 respectively.

Sequencher 4.6 (GeneCodes, Ann Arbor, MI, USA) was used to assemble and edit the DNA sequence. Sequences were aligned in MAFFT 7 (https://mafft.cbrc.jp/alignment/server/) using the “G-INS-I” strategy and manually adjusted in BioEdit (Hall 1999). The sequence alignment was deposited in TreeBase (submission ID 25382). Sequences of *Palifer verecundus* (G. Cunn.) Stalpers & P.K. Buchanan and *Xylodon asperus* (Fr.) Hjortstam & Ryvarden obtained from GenBank were used as outgroups to root trees following Yurchenko et al. (2017) in Fig. 1.

Maximum parsimony (MP), Maximum Likelihood (ML) and Bayesian Inference (BI) analyses were applied to the ITS dataset sequences. Approaches to phylogenetic analyses followed Wu et al. (2018) and Zhu et al. (2019) the tree construction procedure was performed in PAUP* version 4.0b10 (Swofford 2002). All characters were equally weighted and gaps were treated as missing data. Trees were inferred using the heuristic search option with TBR branch swapping and 1000 random sequence additions. Max-trees were set to 5000, branches of zero length were collapsed and all most-parsimonious trees were saved. Clade robustness was assessed using bootstrap (BT) analysis with 1000 replicates (Felsenstein 1985). Descriptive tree statistics tree length (TL), consistency index (CI), retention index (RI), rescaled consistency index (RC) and homoplasy index (HI) were calculated for each most-parsimonious tree generated.

Sequences were also analyzed using Maximum Likelihood (ML) ML analysis was conducted with RAxML-HPC2 through the Cipres Science Gateway (www.phylo.org; Miller et al. 2009). Branch support (BS) for ML analysis was determined by 1000 bootstrap replicates and evaluated under the gamma model.

MrModeltest 2.3 (Nylander 2004) was used to determine the best-fit evolution model for the data set for Bayesian Inference (BI). Bayesian Inference was performed with MrBayes 3.1.2 with a general time reversible (GTR) model of DNA substitution and a gamma distribution rate variation across sites (Ronquist and Huelsenbeck 2003). Four Markov chains were used in each of 2 runs from random starting trees for 600,000 generations, with trees and parameters sampled every 100 generations. The first quarter of generations were discarded as burn-in. A majority rule consensus tree of all remaining trees and posterior probabilities was calculated. Branches that received bootstrap support for maximum likelihood (BS) ≥75%, maximum parsimony (BP) ≥75%, and Bayesian posterior probabilities (BPP) ≥0.95 were considered significantly supported.

### Phylogeny results

The ITS dataset (Fig. 1) included sequences from 39 fungal specimens representing 18 species. The dataset had an aligned length of 608 characters, of which 277 characters were constant and 242 parsimony-informative. MP analysis yielded 8 equally parsimonious trees (TL = 978, CI = 0.523, HI = 0.478, RI = 0.738, RC = 0.385). The best-fit model for ITS alignment estimated and applied in the BI was GTR+I+G. At the end of the BI runs, the average standard deviation of split frequencies was 0.008676. The tree topology obtained by BI and ML was similar to the MP one.

The phylogenetic tree (Fig. 1) demonstrated that all samples grouped into the *Lyomyces* in the present study. *Lyomyces bambusinus* grouped with *L. sambuci*. *Lyomyces cremeus* formed a monophyletic lineage and then grouped with *L. microfasciculatus* (Yurchenko & Sheng H. Wu) Riebesehl & Langer. *Lyomyces macrosporus* was sister to *L. allantosporus* Riebesehl, Yurchenko & Langer. *Lyomyces wuliangshanensis* was closely related to *L. mascarensis* Riebesehl, Yurchenko & Langer.

## Taxonomy

### Lyomyces bambusinus

Taxon classificationFungiPolyporalesMeruliaceae

C.L. Zhao sp. nov.

EB36778F-AB0F-5D73-ABC9-247BCDC0B85A

834036

Figs 2 , 6

#### Holotype.

China. Yunnan Province: Puer, Zhenyuan County, Heping Town, Ailaoshan National Nature Reserve, on dead bamboo, 11 January 2018, CLZhao 4831 (SWFC).

#### Etymology.

The epithet *bambusinus* (Lat.): refers to the occurrence on bamboo.

#### Basidiomata.

Annual, resupinate, ceraceous when fresh, becoming brittle and cracking upon drying, up to 20 cm long and 8 cm wide, 100–200 µm thick. Hymenial surface colliculose to tuberculate, white to cream when fresh, turning cream to buff upon drying. Margin narrow, concolorous with hymenial surface.

#### Hyphal system.

Monomitic; generative hyphae with clamp connections, hyaline, thick-walled, branched, 2.5–3.9 µm in diameter, IKI–, cyanophilous; tissues unchanged in KOH. Numerous crystals present among hyphae.

#### Hymenium.

Two kinds of cystidia: 1) capitate, hyaline, thin-walled, 35–55 × 4–7 µm, smooth or slightly encrusted; 2) tapering, hyaline, thin-walled, 40–65 × 4–5.5 µm, smooth or slightly encrusted; cystidioles present, hyaline, thin-walled, 12–17 × 2–3 µm. Basidia clavate, constricted, thin-walled, with four sterigmata and a basal clamp connection, 16.5–35 × 3.5–7 µm.

#### Spores.

Basidiospores broadly ellipsoid, hyaline, slightly thick-walled, smooth, IKI–, cyanophilous, guttulate, (4.5–)4.7–5.9 (–6.2) × (3.4–)3.7–4.6(–4.8) µm, L = 5.31 µm, W = 4.19 µm, Q = 1.23–1.3 (n = 120/4).

#### Ecology and distribution.

On dead bamboo, causing a white rot. China.

#### Additional specimens examined.

China. Yunnan Province: Puer, Jingdong County, Wuliangshan National Nature Reserve, on dead bamboo, 3 October 2017, CLZhao 3675; Zhenyuan County, Heping Town, Ailaoshan National Nature Reserve, on dead bamboo, 11 January 2018, CLZhao 4808, CLZhao 4840 (SWFC).

**Figure 2.**
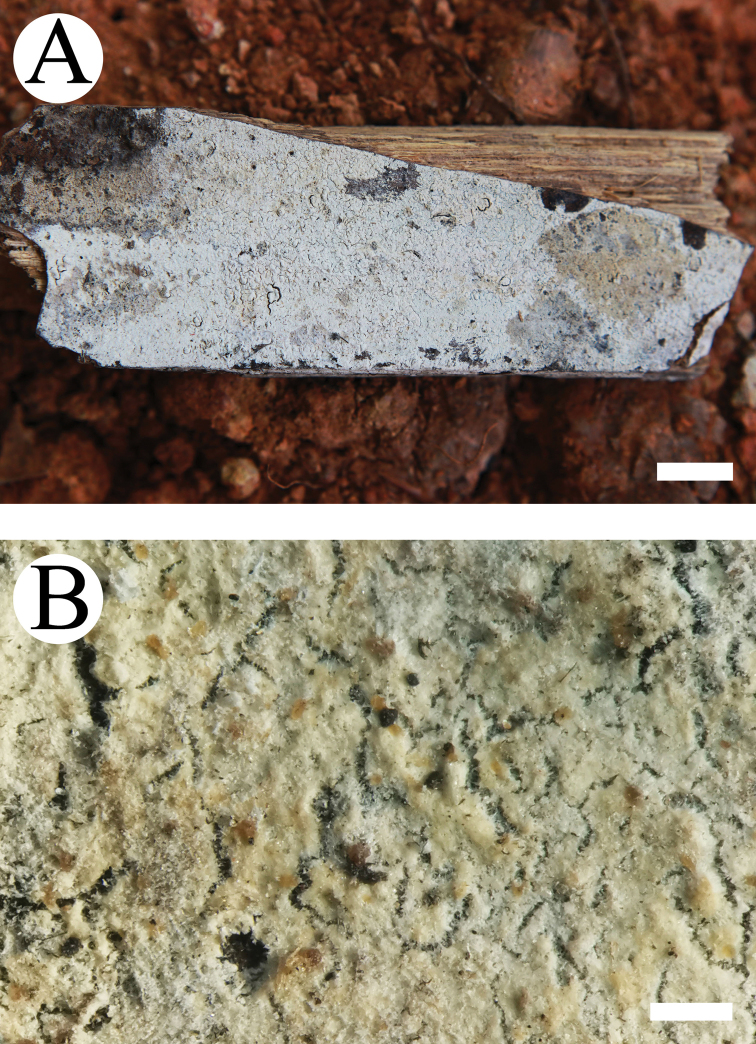
Basidiomata of *Lyomyces bambusinus* (holotype). Scale bars: 1 cm (**A**); 5 mm (**B**).

### Lyomyces cremeus

Taxon classificationFungiPolyporalesMeruliaceae

C.L. Zhao sp. nov.

C9612282-0541-5753-91E4-857E11CA0545

834037

Figs 3 , 7

#### Holotype.

China. Yunnan Province: Jingdong County, Taizhong Town, Ailaoshan Ecological Station, on fallen branch of angiosperm, 23 August 2018, CLZhao 8295 (SWFC).

#### Etymology.

The epithet *cremeus* (Lat.): refers to the cream hymenial surface.

#### Basidiomata.

Annual, resupinate, ceraceous when fresh, becoming membranaceous upon drying, up to 13 cm long and 5 cm wide, 50–100 µm thick. Hymenial surface smooth, pale cream when fresh, turn cream upon drying. Margin narrow, white to cream.

#### Hyphal system.

Monomitic; generative hyphae with clamp connections, hyaline, thick-walled, branched, 3–5 µm in diameter, IKI–, cyanophilous; tissues unchanged in KOH. Numerous crystals present among hyphae.

#### Hymenium.

Two kinds of cystidia: 1) capitate, hyaline, thin-walled, 20–40 × 3–5 µm, smooth or slightly encrusted; 2) tapering, hyaline, thin-walled, 18–35 × 3–4.5 µm, smooth or slightly encrusted; cystidioles present, hyaline, thin-walled, 15–20 × 2.5–4 µm. Basidia clavate, with four sterigmata and a basal clamp connection, 9–18.5 × 3–6 µm.

#### Spores.

Basidiospores ellipsoid, hyaline, thin-walled to slightly thick-walled, smooth, IKI–, cyanophilous, guttulate, 4.5–5.6(–5.8) × 3.3–4.3(–4.5) µm, L = 5.01 µm, W = 3.94 µm, Q = 1.25–1.3 (n = 90/3).

#### Ecology and distribution.

Lignicolous, causing a white rot. China.

#### Additional specimens examined.

China. Yunnan Province: Yuxi, Xinping County, Shimenxia Forestry Park, on fallen branch of angiosperm, 21 August 2017, CLZhao 2812; Puer, Jingdong County, Wuliangshan National Nature Reserve, on fallen branch of angiosperm, 5 October 2017, CLZhao 4138 (SWFC).

**Figure 3.**
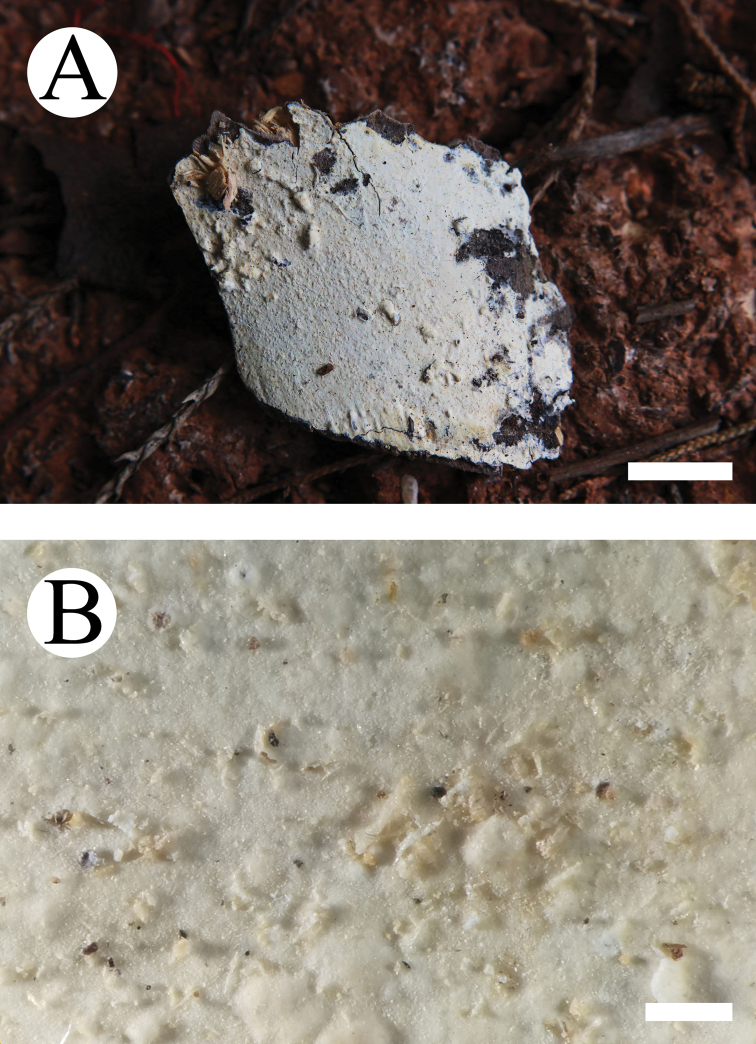
Basidiomata of *Lyomyces cremeus* (holotype). Scale bars: 1 cm (**A**); 5 mm (**B**).

### Lyomyces macrosporus

Taxon classificationFungiPolyporalesMeruliaceae

C.L. Zhao sp. nov.

BDE71C96-59A9-514A-9643-06C9AC9BF430

834038

Figs 4 , 8

#### Holotype.

China. Yunnan Province: Puer, Jingdong County, Taizhong Town, Ailaoshan National Nature Reserve, on fallen branch of angiosperm, 24 August 2018, CLZhao 8605 (holotype in SWFC).

#### Etymology.

The epithet *macrosporus* (Lat.): refers to the larger basidiospores.

#### Basidiomata.

Annual, resupinate, subceraceous when fresh, becoming pruinose upon drying, up to 22 cm long and 3 cm wide, 100–200 µm thick. Hymenial surface reticulate, cream when fresh, turning cream to buff upon drying. Margin narrow, white to buff.

#### Hyphal system.

Monomitic; generative hyphae with clamp connections, hyaline, thick-walled, branched, 2.5–4 µm in diameter, IKI–, cyanophilous; tissues unchanged in KOH. Numerous crystals present among hyphae.

#### Hymenium.

Three kinds of cystidia: 1) capitate, hyaline, thin-walled, 19–35 × 3–7 µm; 2) tapering, hyaline, thin-walled, 13–20 × 2.5–4 µm; 3) moniliform, hyaline, thin-walled, 15–22 × 4.5–6 µm; fusoid cystidioles present, hyaline, thin-walled, 15–20 × 2.5–4 µm. Basidia subclavate to clavate, constricted, hyaline, thin-walled, with four sterigmata and a basal clamp connection, 22.2–38 × 4.5–7 µm.

#### Spores.

Basidiospores ellipsoid, hyaline, slightly thick-walled, smooth, IKI–, cyanophilous, guttulate, (6.4–)6.7–8.9(–9.1) × 4.4–5.4(–5.7) µm, L = 7.84 µm, W = 4.93 µm, Q = 1.48–1.8 (n = 120/4).

#### Ecology and distribution.

Lignicolous, causing a white rot. China.

#### Additional specimens examined.

China. Yunnan Province: Puer, Jingdong County, Taizhong Town, Ailaoshan National Nature Reserve, on fallen branch of angiosperm, 4 October 2017, CLZhao 3951; Wuliangshan National Nature Reserve, on fallen branch of angiosperm, 6 October 2017, CLZhao 4516, CLZhao 4531 (SWFC).

**Figure 4.**
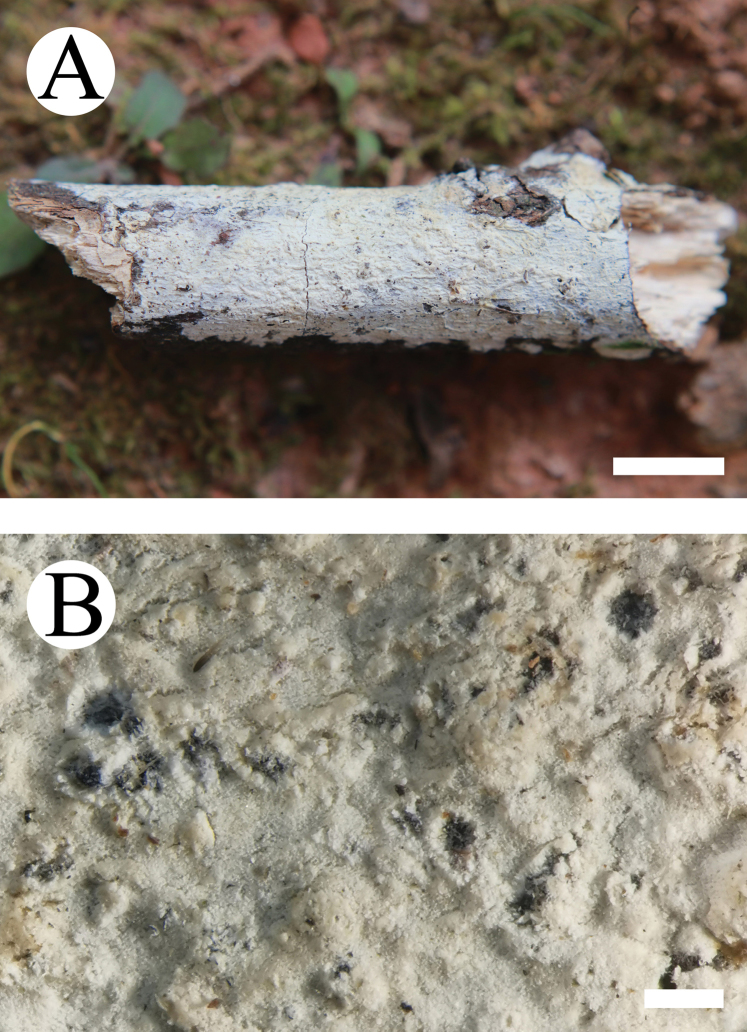
Basidiomata of *Lyomyces macrosporus* (holotype). Scale bars: 1 cm (**A**); 5 mm (**B**).

### Lyomyces wuliangshanensis

Taxon classificationFungiPolyporalesMeruliaceae

C.L. Zhao sp. nov.

0199474D-E47D-572B-B13B-F9D111501B5C

834039

Figs 5 , 9

#### Holotype.

China. Yunnan Province: Puer, Jingdong County, Wuliangshan National Nature Reserve, on fallen branch of angiosperm, 5 October 2017, CLZhao 4167 (SWFC).

#### Etymology.

The epithet *wuliangshanensis* (Lat.): refers to the locality (Wuliangshan) of the type specimens.

#### Basidiomata.

Annual, resupinate, subcoriaceous when fresh, becoming coriaceous upon drying, up to 15 cm long and 5 cm wide, 50–150 µm thick. Hymenial surface smooth to more or less tuberculate, white to cream when fresh, turning cream to buff upon drying. Margin narrow, concolorous with hymenial surface.

#### Hyphal system.

Monomitic; generative hyphae with clamp connections, hyaline, thick-walled, branched, 2–3 µm in diameter, IKI–, cyanophilous; tissues unchanged in KOH. Numerous crystals present among hyphae.

#### Hymenium.

Two kinds of cystidia: 1) capitate, hyaline, thin-walled, 22–37 × 3–6 µm; 2) tapering, hyaline, thin-walled, 21–35 × 4–6.5 µm; fusoid cystidioles present, hyaline, thin-walled, 16–21 × 2.5–3.5 µm. Basidia clavate, hyaline, thin-walled, with four sterigmata and a basal clamp connection, 12–20 × 3–4.3 µm.

#### Spores.

Basidiospores ellipsoid, hyaline, slightly thick-walled, smooth, IKI–, cyanophilous, guttulate, (3.3–)3.5–5.3(–5.5) × 2.8–4(–4.2) µm, L = 4.3 µm, W = 3.56 µm, Q = 1.22–1.31 (n = 120/4).

#### Ecology and distribution.

Lignicolous, causing a white rot. China.

#### Additional specimens examined.

China. Yunnan Province: Puer, Jingdong County, Wuliangshan National Nature Reserve, on angiosperm trunk, 5 October 2017, CLZhao 4108, CLZhao 4144; on angiosperm stump, 5 October 2017, CLZhao 4206; on fallen branch of angiosperm, 6 October 2017, CLZhao 4475 (SWFC).

**Figure 5.**
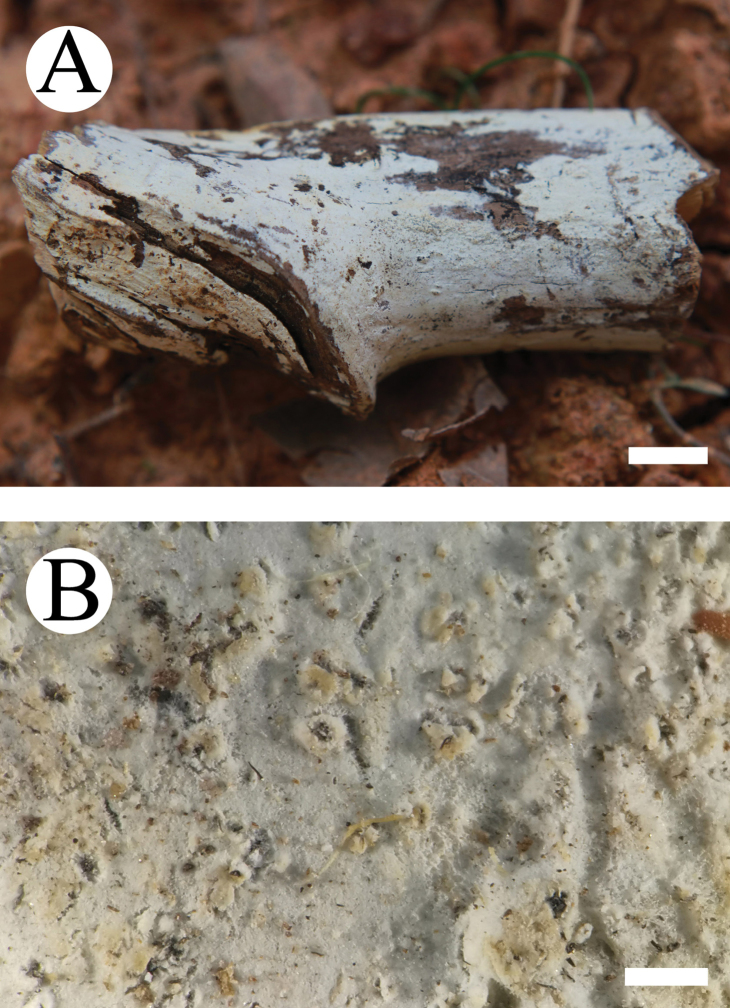
Basidiomata of *Lyomyces wuliangshanensis* (holotype). Scale bars: 1 cm (**A**); 5 mm (**B**).

**Figure 6.**
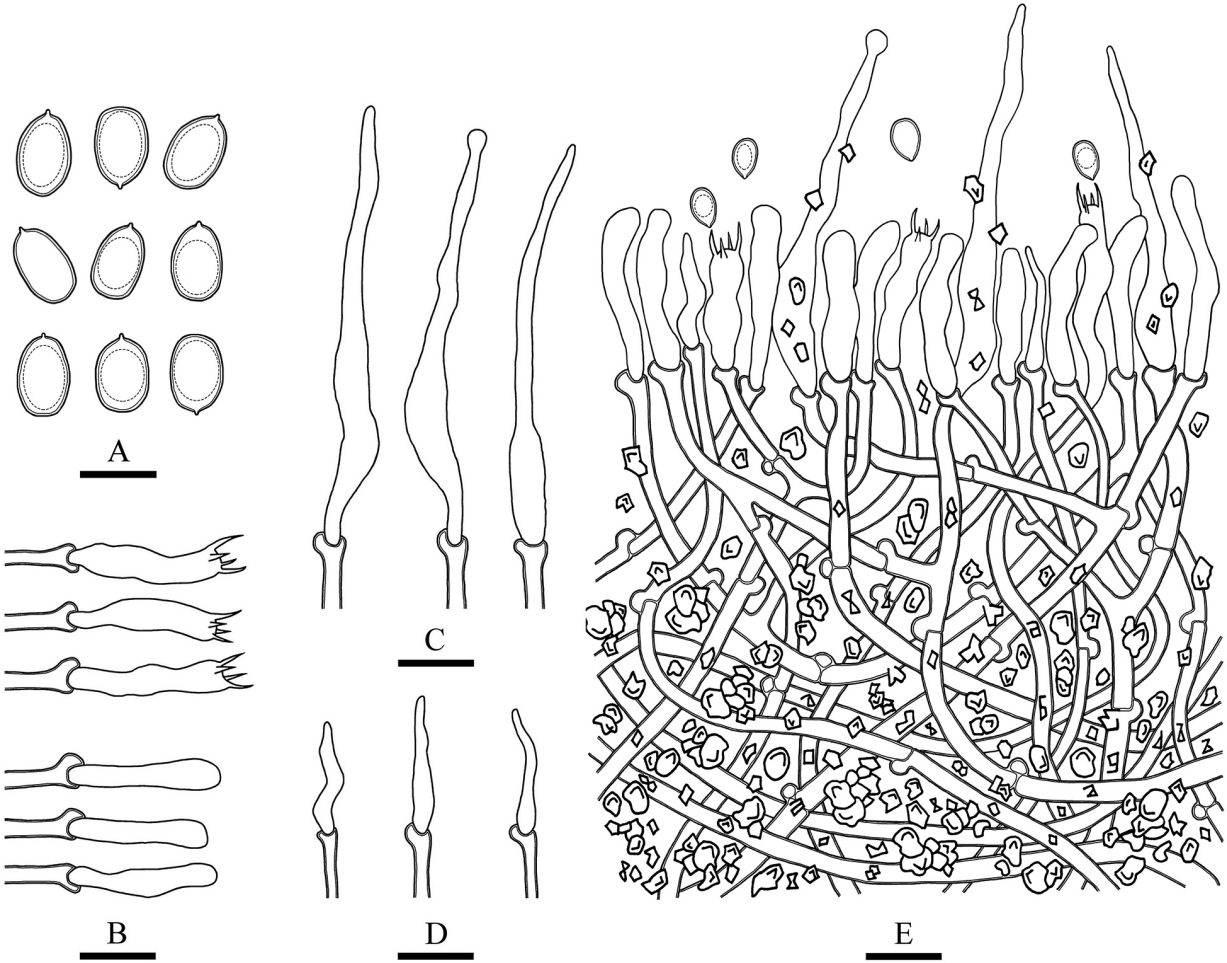
Microscopic structures of *Lyomyces bambusinus* (drawn from the holotype) **A** basidiospores **B** basidia and basidioles **C** cystidia **D** cystidioles **E** a cross section of basidiomata. Scale bars: 5 μm (**A**); 10 μm (**B–E**).

**Figure 7.**
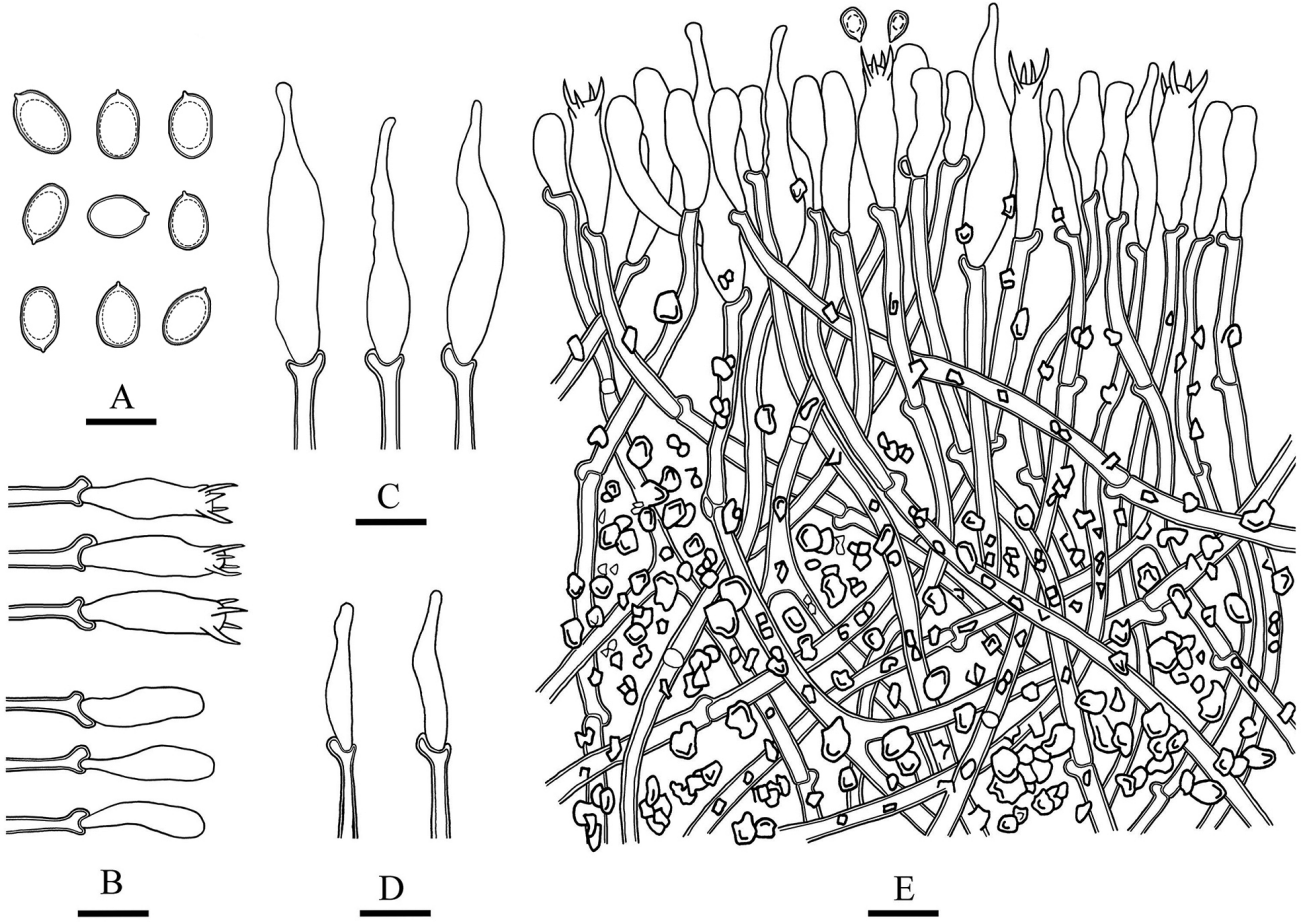
Microscopic structures of *Lyomyces cremeus* (drawn from the holotype) **A** basidiospores **B** basidia and basidioles **C** cystidia **D** cystidioles **E** a cross section of basidiomata. Scale bars: 5 μm (**A**); 10 μm (**B–E**).

**Figure 8.**
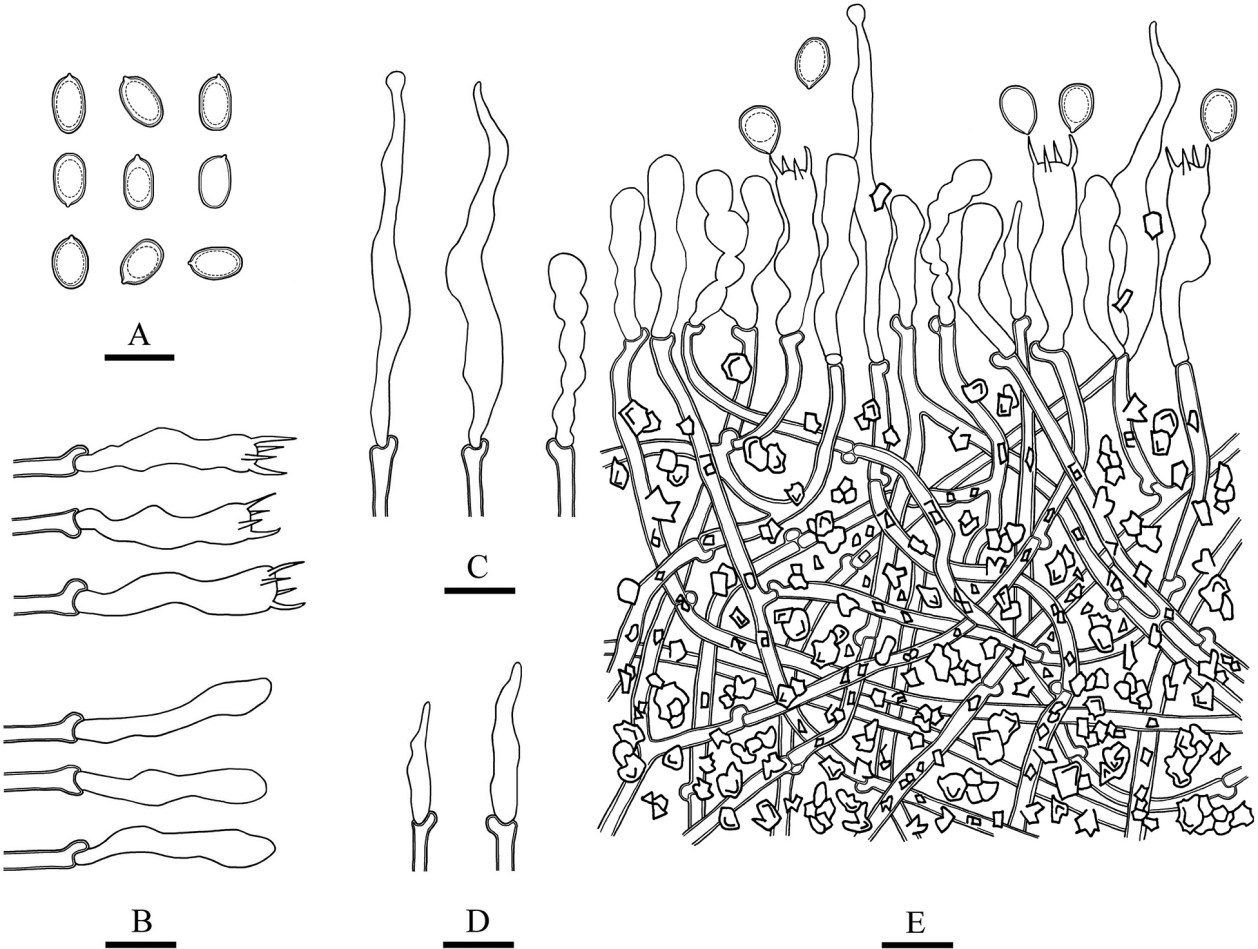
Microscopic structures of *Lyomyces macrosporus* (drawn from the holotype) **A** basidiospores **B** basidia and basidioles **C** cystidia **D** cystidioles **E** a cross section of basidiomata. Scale bars: 10 μm (**A–E**).

**Figure 9.**
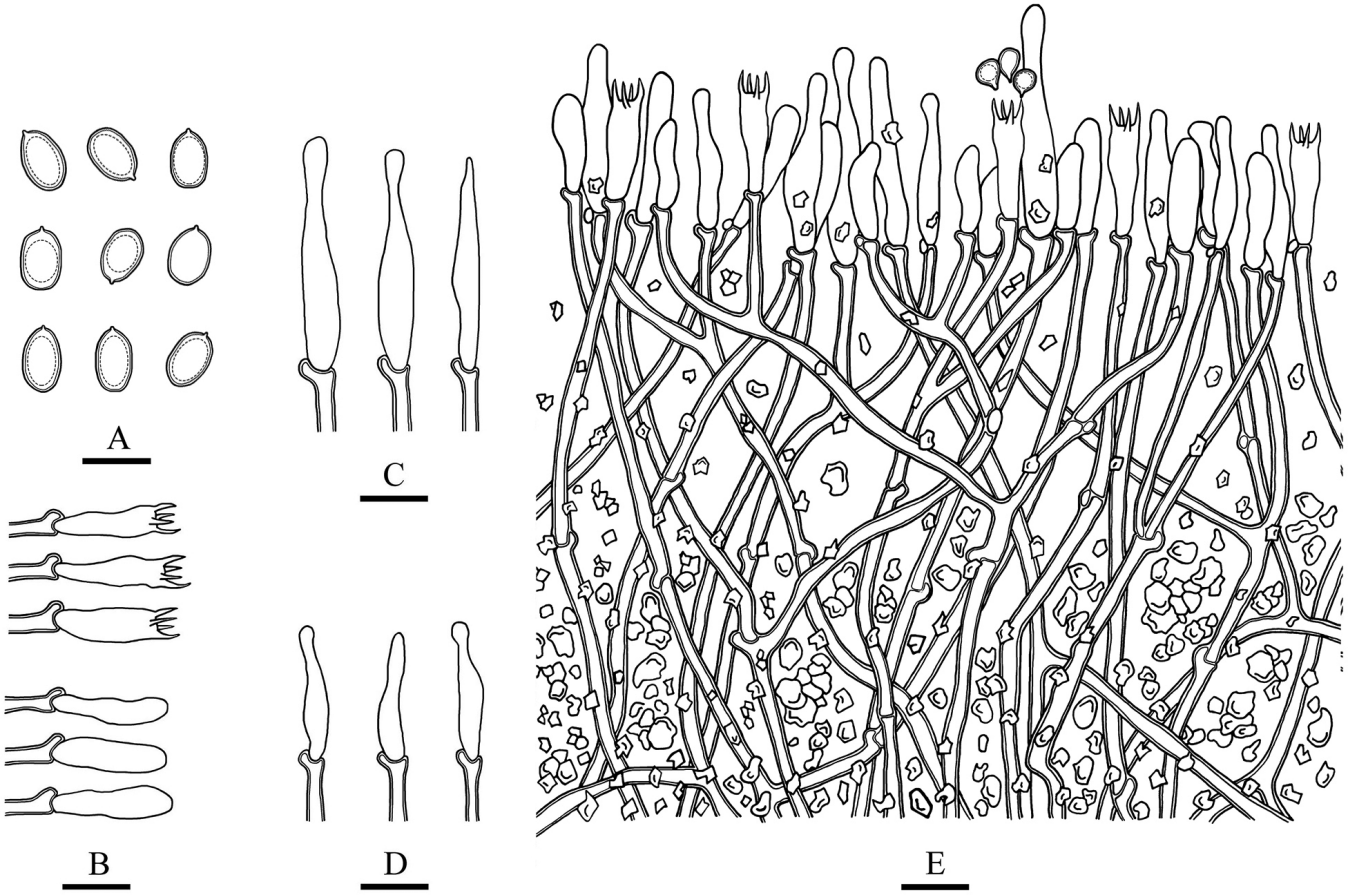
Microscopic structures of *Lyomyces wuliangshanensis* (drawn from the holotype) **A** basidiospores **B** basidia and basidioles **C** cystidia **D** cystidioles **E** a cross section of basidiomata. Scale bars: 5 μm (**A**); 10 μm (**B–E**).

## Discussion

Miettinen et al. (2016) analyzed a phylogenetic classification in Phanerochaetaceae (Polyporales, Basidiomycota) and showed that the macromorphology of basidiomata and hymenophore construction did not reflect monophyletic groups. The phylogeny we obtained (Fig. 1) shows that the macromorphological and micromorphological characters are not consistent with monophyletic groups.

In our phylogeny, *Lyomyces bambusinus* was sisiter to *L. sambuci*, but morphologically *L. sambuci* differs from *L. bambusinus* by having ellipsoid to oblong, narrower basidiospores (4.5–6 × 3–3.5 µm, Yurchenko et al. 2017). *Lyomyces cremeus* formed a monophyletic lineage with strong supports (100% BS, 100% BP, 1.00 BPP; Fig. 1) and then was sister to a clade comprised of *L. microfasciculatus*, *L. mascarensis*, and *L. wuliangshanensis*. However *L. microfasciculatus* differs in odontioid hymenophore and presence of minute peg-like fascicles of hyphae (Yurchenko and Wu 2014). *Lyomyces macrosporus* was sister to *L. allantosporus*, but morphologically *L. allantosporus* differs in having porulose hymenophore and suballantoid to allantoid, narrower basidiospores (7–9 × 3–3.8 µm, Yurchenko et al. 2017). *Lyomyces wuliangshanensis* grouped closely with *L. mascarensis*, but *L. mascarensis* differs from *L. wuliangshanensis* by having thin-walled generative hyphae and presence of capitate cystidia (17–38 × 3.5–6 µm, Yurchenko et al. 2017).

Five *Lyomyces* species were reported from China prior to this study, *Lyomyces albus* (Sheng H. Wu) Riebesehl & Langer, *L. capitatocystidiatus* (H.X. Xiong, Y.C. Dai & Sheng H. Wu) Riebesehl & Langer, *L. microfasciculatus*, *L. sambuci* and *L. tenuissimus* (Yurchenko & Sheng H. Wu) Riebesehl & Langer. *Lyomyces albus* differs from four new species by its odontioid hymenophore (Riebesehl and Langer 2017); *L. capitatocystidiatus* by grandinioid hymenophore with arachnoid-farinaceous hymenial surface (Xiong et al. 2009); *L. microfasciculatus* by minutely odontioid hymenial surface and small emerging fascicles of flexuous hyphae (Yurchenko and Wu 2014); *L. tenuissimus* by much thinner basidiomata and non-encrusted, subicular hyphae, shorter cystidia and shorter basidia (Yurchenko et al. 2013).

*Hyphodontia* s.l. is an extensively studied group of Hymenochaetales (Dai 2012; Viner et al. 2018; Riebesehl et al. 2019), but the Chinese species diversity is still not well known, especially in subtropical and tropical areas. The four new *Lyomyces* species here described are from the subtropics.

## Supplementary Material

XML Treatment for Lyomyces bambusinus

XML Treatment for Lyomyces cremeus

XML Treatment for Lyomyces macrosporus

XML Treatment for Lyomyces wuliangshanensis
